# Production and Easy One-Step Purification of Bluetongue Recombinant VP7 from Infected Sf9 Supernatant for an Immunoenzymatic Assay (ELISA)

**DOI:** 10.1007/s12033-020-00282-8

**Published:** 2020-10-19

**Authors:** S. Ulisse, M. Iorio, G. Armillotta, C. Laguardia, L. Testa, S. Capista, P. Centorame, S. Traini, A. Serroni, F. Monaco, M. Caporale, M. T. Mercante, M. Di Ventura

**Affiliations:** Istituto Zooprofilattico Sperimentale dell’Abruzzo e del Molise “G. Caporale”, Teramo, Italy

**Keywords:** BTV, Recombinant VP7, Baculovirus, Supernatant, Affinity chromatography, ELISA

## Abstract

Bluetongue (BT) is non-contagious, vector-borne viral disease of domestic and wild ruminants, transmitted by midges (*Culicoides* spp.) and is caused by Bluetongue virus (BTV). BTV is the type species of the *Orbivirus* genus within the *Reoviridae* family and possesses a genome consisting of 10 double-stranded RNA segments encoding 7 structural and 4 nonstructural proteins. Viral Protein 7 (VP7) is the major sera group-specific protein and is a good antigen candidate for immunoenzymatic assays for the BT diagnosis. In our work, BTV-2 recombinant VP7 (BTV-2 recVP7), expressed in *Spodoptera frugiperda* (Sf9) cells using a baculovirus system, was produced and purified by affinity chromatography from the supernatant of infected cell culture. The use of the supernatant allowed us to obtain a high quantity of recombinant protein with high purity level by an easy one-step procedure, rather than the multistep purification from the pellet. RecVP7-BTV2 was detected using a MAb anti-BTV in Western blot and it was used to develop an immunoenzymatic assay.

## Introduction

Bluetongue virus (BTV) is a member of the *Orbivirus* genus of the family *Reoviridae* [1, 2] and infects sheep, cattle and wild ruminants. BT has evolved in the past ten years from an exotic disease, restricted to warm Sub-Saharian climates, to a more widespread disease, with the potential of becoming endemic in new areas with temperate climates [3, 4], causing high economic impact on the international livestock industry. BTV has a double stranded RNA (dsRNA) genome formed by 10 segments encoding 7 structural and 4 non-structural proteins [5–8]. The BTV virion consists of a triple-layered icosahedral protein capsid [7, 9], that gives the name to the species. The outer capsid layer of the virion is formed by VP2 and VP5 that together elicit virus neutralizing antibodies [10]. To date, at least 27 serotypes have been identified worldwide [11–14]. The viral internal core is composed of two layers, constituted by VP3 (sub core) and VP7 (core surface layer) [6, 7] the latter identifies the BTV serogroup [15, 16]. BTV core also contains the three minor enzymatic core proteins, VP1 (RNA-dependent RNA polymerase), VP4 (capping enzyme and transmethylase), and VP6 (RNA-dependent ATPase and helicase). VP7, a ~ 40 kDa protein arranged as trimers to form the BTV core surface [5–8], is conserved within all the different BTV serotypes and is used for serodiagnostic purpose to detect infected animals [15, 16]. A variety of recombinant DNA methods have been used to express BTV VP7 protein [9, 17–21] and among these the baculovirus expression system has been successfully used to reach a high yield production of the biologically active recombinant VP7 protein [9, 18]. This method is preferred because it is able to overcome the protein solubility problems that usually occur when prokaryotic expression systems are used [19, 20, 22]. In this work, we describe a recombinant BTV VP7 production method using a baculovirus expression system and a subsequent purification method of the protein by immobilized metal affinity chromatography (IMAC). As reported in literature [18, 23–25], recombinant VP7 *Orbiviruses* proteins, such as BTV and EHDV, were usually purified from infected cell pellet or they were directly used for the development of immunoassays from infected cell culture supernatant-pellet without further purification [26, 27]. We used both supernatant and pellet of infected Sf9 cells as biological sources of BTV recVP7, that were purified with different experimental conditions, in order to assess the most suitable method. Recombinant VP7, purified from both supernatant and pellet, was further evaluated with a c-ELISA using in house developed specific monoclonal antibody.

## Materials and Methods

### Viruses and Cells

Bluetongue Virus serotype 2 (BTV-2) was selected from a panel of BTV field strains isolated during an outbreak in Italy in 2000 (Genebank accession number JN255862) and it was used to infect VERO cells (ATCC® CCL-81). VERO cells were grown in MEM with Earle's Salts and l-Glutamine (Biowest) and 10% Fetal Bovine Serum (FBS) (Sigma-Aldrich) at 37 °C, 5% CO_2_. Virus particles were purified from infected cells supernatant using methods already described in literature [28].

Propagation of recombinant baculovirus and expression of BTV-2 recVP7 was performed in Sf9 cells (ECACC 05011001), maintained in suspension cultures using Sf-900II SFM medium (Gibco) in vent cap Erlenmeyer shaker flasks (Corning) at 27 °C and 110 rpm speed. Sf9 Easy Titer cells, gently provided from Ralph F. Hopkins, Protein Expression Laboratory, Advance Technology Program, SAIC-Frederick, National Cancer Institute at Frederick, MD, USA, were cultured and used according to the method described in Hopkins and Esposito [29].

### Generation of Recombinant Plasmid and Transfer Vector

Total RNA was extracted from the infected cell monolayer using the High Pure Viral Nucleic Acid Kit (Roche). The full-length cDNA of Seg-7, coding VP7 protein, was obtained by retrotranscription with Superscript II RTase (Invitrogen) using random primers and amplified using Pfu turbo polymerase (Stratagene). Primer used for cDNA amplification were designed from reference sequence of BTV serotype 2 (Genbank accession number JN255868.1), including restriction site sequences specific for KpnI and XhoI (Roche):

FwKpnIvp7_2_5′-ATAT*GGTACC*ACGACACTATCGCGGCAAGAG-3′.

RwXhoIvp7_2_5′-ACAC*CTCGAG*TTACTACACATAAGCGGCGC-3′.

The Seg-7 cDNA was cloned into pCR-XL-TOPO (Invitrogen) and sub-cloned into pENTR1A Dual Selection (Invitrogen) according manufacturer instructions. We obtained the pENTR1A-BTV2-VP7 that was verified by restriction analysis and sequenced using the BigDye X Terminator kit and ABI PRISM 3100 sequencer (Applied Biosystems). pENTR1A BTV-2-VP7 was subsequently used to perform homologous recombination reaction to transfer the gene of interest into the N-terminal BaculoDirect™ Vector (Invitrogen), obtaining the recombinant *Autographa californica multiple nucleopolyhedrovirus* (rAcMNPV_BTV-2_VP7) containing V5 and His tag in N-terminal on BTV-2 VP7, according manufacturer instructions.

### Recombinant Baculovirus Production

Sf9 cells were transfected using Cellfectin® II Reagent (Invitrogen, USA) with the recombinant baculovirus rAcMNPV_BTV-2_VP7 vector described above, according to the manufacturer instructions (BaculoDirect™ Baculovirus Expression System, ThermoFisher, USA). Briefly, 72 h post-transfection the supernatant, viral stock P1, was collected and stored at − 80 °C. Aliquots of both infected crude Sf9 cell lysate and unpurified infected Sf9 cell culture supernatant, from here on defined “pellet “ and “supernatant” respectively, were tested in Western blot using anti-V5 HRP antibody. The viral stock P1 was propagated in Sf9 cells in 125 mL shaker flasks at 1.5 × 10^6^ cells/mL to generate viral stocks P2 and P3, using Multiplicity of Infection (MOI) of 0.1. P2 and P3 were collected 72 h post-infection (p.i.) and titrated with a novel cell line, Sf9 Easy Titer cells [29] using end-point dilution method. Sf9 Easy Titer cells were stably tranfected with plasmid DNA containing the enhanced green fluorescent protein (eGFP) gene under the control of the baculovirus polyhedrin promoter and they turn green when are infected whit baculovirus due to activation of the polyhedrin promoter/eGFP complex. Briefly, 100 µL per well of a suspension of Sf9 Easy Titer cells (8 × 10^5^ cells/mL) were added to a 96-well microtiter plate. Then, serial dilutions of a virus stock was prepared and inoculated onto Sf9 Easy Titer cell culture. After five days, the number of cells that were infected is then determined for each virus dilution and this is assessed using a fluorescence microscope by scoring GFP-positive wells. The viral titer value was calculated with Reed e Müench method. The result was obtained in TCID_50_/mL (median tissue culture infectious dose) that multiplied for the conversion factor 0.7 gives us the corresponding value in PFU/mL.

### Expression and Isolation of BTV-2 recVP7

The optimal MOI and Time of Harvest (TOH), to maximize the recombinant protein expression, were defined through a small-scale experiment. Briefly, 50 mL of Sf9 cell suspension with a density of 3.5 × 10^6^ ± 0.1 cells/mL, were infected with rAcMNPV BTV-2_VP7_P3 at 0.001, 0.01, 0.1 and 1 MOI. The infected cells were collected at 0, 24, 48, 72, 96 and 120 h p.i. and were evaluated for cell density, cell viability and cell diameter by CountessTM automated cell counter (Invitrogen, USA), in order to evaluate cellular changes as a result of a baculovirus infection.

Viral titration was performed on all the samples as described above. VP7 expression was assessed by SDS-PAGE and Western blot using an anti-V5 HRP antibody (Invitrogen). Production of the BTV-2 recVP7 was scaled-up to 1.5 L of Sf9 cell culture.

Infected cells were harvested by centrifugation at 3500×*g* for 10 min at 4 °C. The cell pellet was rinsed with phosphate-buffered saline pH 7.5 (PBS) and stored at − 80 °C until purification. The supernatant was sterile-filtered and stored at 4 °C until the chromatographic purification.

### BTV-2 recVP7 Ammonium Sulphate Precipitation

BTV-2 recVP7 was precipitated from the pellet obtained as previously described according to the protocol of Luo and Sabara (2008), with minor modifications. Briefly, Sf9 cell culture pellet was thawed and washed with PBS, lysed in 10 mM Tris–HCl (pH 7.5) containing 0.5% NP40, protease inhibitors cocktail (Roche), 3.3 M l-Arginine hydrochloride [30–33] and incubated for 3 h on ice.

Cell debris and nucleic DNA were removed by centrifugation at 3500×*g* for 10 min. A saturated solution of ammonium sulphate, prepared in 100 mM Tris–HCl pH 7.5, was added to the cytoplasmic cell extract to a final saturation of 20% (v/v) and incubated with gentle agitation overnight (O.N.) at 4 °C. The precipitated proteins were collected by centrifugation at 16,000×*g* for 10 min and resuspended in 10 mM Tris–HCl solution, pH 7.5.

A sample was collected for further analyses using Western blot and the remaining extracted BTV-2 recVP7 protein was stored at 4 °C until the next purification step using IMAC.

### BTV-2 recVP7 Purification by IMAC

L-Arginine hydrochloride 0.2 M was added to the supernatant [30–33] and incubated with gentle agitation at room temperature (R.T.) for 1 h. Supernatant and pellet containing BTV-2 recVP7 were loaded separately on a HisTrap excel column (GE Healthcare), in a fully automated manner using an AKTAPurifier 100 instrument (GE Healthcare). Purification was conducted according to manufacturer instructions. Briefly, the column was equilibrated with 20 mM sodium phosphate, 500 mM sodium chloride and 0.2 M l-Arginine hydrochloride. The column was washed with 20 mM imidazole before the elution step performed with buffer containing 20 mM sodium phosphate, 500 mM sodium chloride, 0.2 M l-Arginine hydrochloride and 250 mM imidazole.

BTV-2 recVP7 purified from both pellet and supernatant were incubated at 4 °C O.N. and centrifuged at 4000×*g* using Amicon Ultra-15 centrifugal Filter Units MWCO 10 KDa. The concentrated BTV-2 recVP7 was recovered and diluted with the same volume of PBS 1×, pH 7.5 containing 0.5% Sarkosyl NL (Sigma-Aldrich), for both samples. The protein concentration was assessed by the Bradford assay and finally, the purity, antigenicity and identity of the BTV-2 recVP7 derived from the cell pellet (pVP7) and the supernatant (sVP7) was checked in SDS-PAGE and Western blot.

### SDS, Semi-native PAGE and Western Blot

All the samples of BTV-2 recVP7 were analyzed by SDS-PAGE. The protein was denatured at 70° with NuPAGE LDS sample buffer and NuPAGE sample reducing agent (Invitrogen) for 10 min and then separated on NuPAGE Novex 4–12% Bis–Tris Gel (Invitrogen). The gel was stained with Bio-safe Coomassie G-250 Stain (Bio-Rad). The P1 and P2 unpurified viral stocks, unpurified recVP7 produced at small-scale and purified recVP7 produced at large scale were analyzed in denaturing conditions; moreover, the samples of recVP7 purified from pellet and supernatant derived from 1.5 L of Sf9 cell culture, were also analyzed in semi-native PAGE thus loaded on NuPAGE gels without prior denaturation treatment.

BTV-2 recVP7 separated by electrophoresis was blotted onto nitrocellulose membranes. Blocking was performed with 5% (w/v) skim milk in PBS containing 0.05% Tween 20 (PBST) at R.T. for 2 h, followed by an incubation with HRP-conjugated MAb anti-BTV [28] and anti-V5 HRP antibody at 1:50,000 and 1:10,000 dilution respectively (Invitrogen) at 4 °C O.N. BTV-2 recVP7 purified from both infected Sf9 pellet and supernatant was also tested with positive sera derived from an experimentally BTV-1 infected bovine and detected with anti-bovine IgG peroxidase antibody (Sigma-Aldrich).The specificity of reaction between MAb anti-BTV [28] and BTV-2 recVP7 from supernatant was evaluated by Western blot using recombinant AHSV-VP7, (IZSAM data not published) and recombinant EHDV-VP7 (purchased from GenScript®).

Novex Sharp Pre-stained Protein Standard was used for molecular weight estimation in SDS-PAGE. Amersham ECL Select Western blotting Detection Reagent (GE Healthcare) and Chemidoc MP Imager (Bio-Rad) were used to acquire images.

### Competitive ELISA

c-ELISA was performed to test the BTV-2 recVP7 purified from pellet and supernatant. 96-well polystyrene plates (Costar) were coated with antigen (100 µL/well), concentrated 1 mg/mL at five different dilutions (1:1000, 1:1500, 1:2000, 1:2500, and 1:3000) in 0.05 M carbonate-bicarbonate buffer (pH 9.6) and incubated at 22 °C O.N. After washing with PBST and blocking with 5% skim milk (Biolife) in PBST at R.T. for 1 h, the plates were washed again and positive serum diluted from 1:32 to 1:128 was added. Undiluted positive and negative sera were used as positive and negative controls respectively.

After incubation at R.T. for 3 h, the plates were washed and different dilutions (1:250,000, 1:300,000 and 1:350,000) of MAb anti-BTV HRP-conjugated [28], concentrated at 8 mg/mL, were added and incubated at R.T. for 1 h. After washing, the plates were developed with 3,3′,5,5′-tetramethyl benzidine substrate (TMB, Surmodics) according to the manufacturer's instructions and incubated at R.T. for 30 min. The reaction was stopped by adding 0.5 N H_2_SO_4_, and optical density (O.D.) was measured at 450 nm.

c-ELISA was also used to verify any cross-reactivity, testing the AHSV and EHDV sera with BTV-2 recVP7 at 5 ng/mL (1:2000 dilution) purified from supernatant. The test was performed using MAb anti-BTV HRP-conjugated at 1:300,000 dilution.

The same protocol and dilutions of recombinant antigen purified from supernatant and MAb anti-BTV HRP-conjugated (1:2000 and 1:300,000 respectively) were used to test a panel of bovine and ovine sera 20 positive and 20 negative, previously characterized by virus-neutralization from OIE Reference Laboratory for Bluetongue, IZSAM. The serotypes tested are two of the serotypes mostly diffused in Italy, in particular the serotypes 1 and 4.

## Results

### Recombinant Baculovirus Generation and BTV-2 recVP7 Expression

**Fig. 1:**
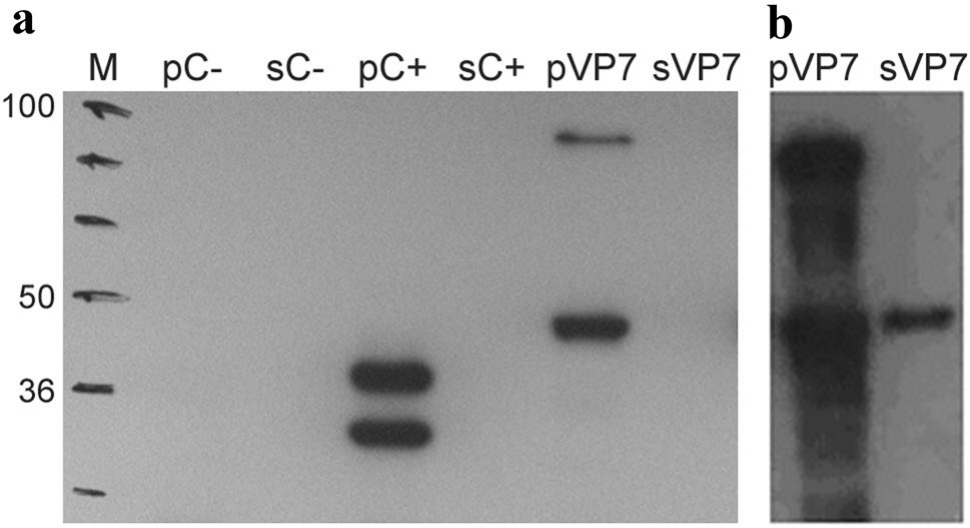
**a**,** b** Western blot detection using anti-V5 antibody of recVP7 from recombinant Baculovirus viral stocks P1 (**a**) and P2 (**b**). PC+ and sC+ were the kit positive control (pENTR/CAT plasmid, 30 kDa); pC− and sC− were the kit negative control (pENTR1A/baculovirus vector); pVP7 and sVP7 were pellet and supernatant samples of rAcMNPV_BTV2_VP7 transfected Sf9 cells

Recombinant baculovirus rAcMNPV_BTV2_VP7 was successfully generated and used to transfect Sf9 cells, in order to obtain P1 viral stock. Pellet and supernatant from P1 were checked for reactivity with anti-V5 HRP antibody (Fig. 1a), that recognized the monomeric and dimeric forms of BTV-2 recVP7 only in the pellet sample.

A different result was obtained analyzing P2 viral stock in Western blot where the BTV-2 recVP7 was present in both pellet and supernatant samples, in form of predominant bands at ~ 40 and ~ 80 kDa in the pellet sample, and a single band at 40 kDa in the supernatant (Fig. 1b).

**Fig. 2:**
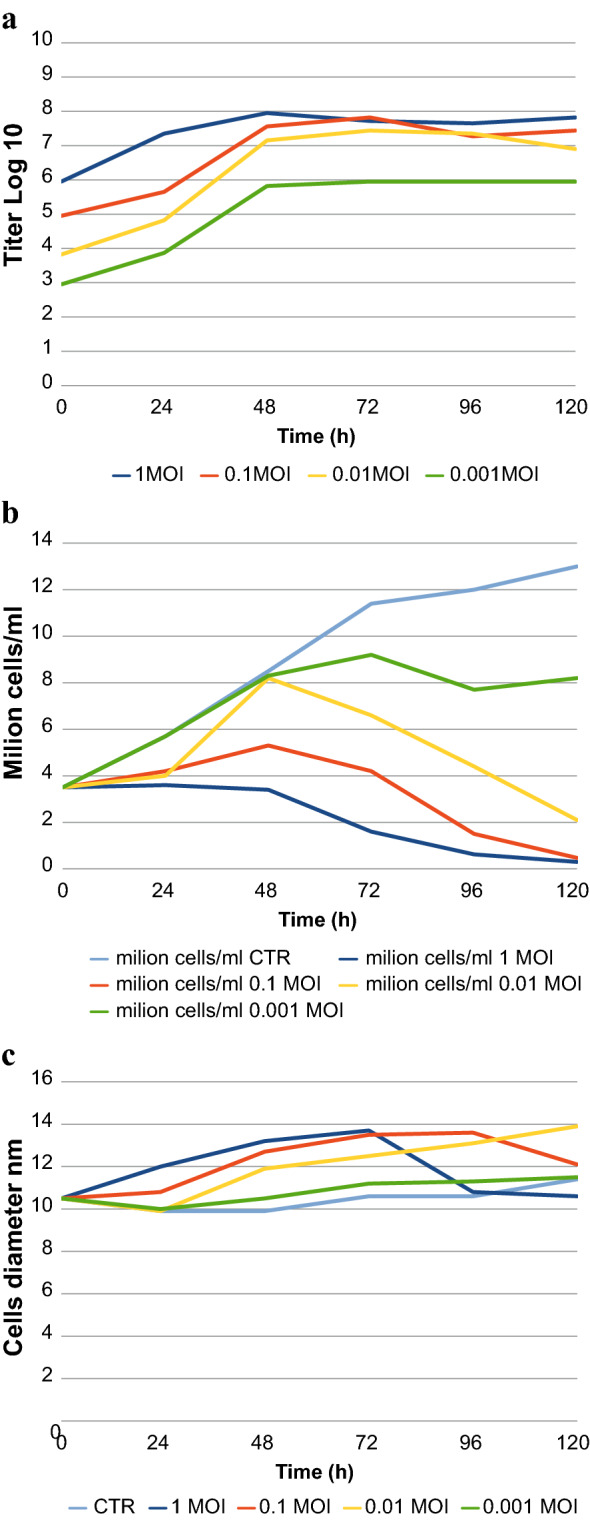
**a** The graph shows the trend over time (0 h, 24 h, 48 h,72 h, 96 h and 120 h) of the viral titer after infection of the Sf9 cells with  0.001 MOI,  0.01 MOI,  0.1 MOI,  1 MOI, of recombinant baculovirus. Titer is expressed in TCID50/mL. **b** The graph shows the trend of cells viability (cells/mL) checked over time (0 h, 24 h, 48 h, 72 h, 96 h and 120 h), after infection of the Sf9 cells with the same MOI of recombinant baculovirus tested in (**a**). **c** The graph shows the Sf9 cells diameter (nm), checked at different timepoints (0 h, 24 h, 48 h, 72 h, 96 h and 120 h), to measure the infection rate of Sf9 by recombinant baculovirus at different MOI

P2 and P3 were titrated used Sf9 Easy Titer cells as described in Materials and Methods and showed a titer of 1 × 10^8.48^ Plaque Forming Unit (PFU)/mL and 1 × 10^7.82^ PFU/mL respectively. Virus preparations, used for the inocula, were also titrated and titers are indicated for time zero (Fig. 2a). The highest viral titers were obtained with MOI 1 harvesting 48 h p.i. and with MOI 0.1 and 72 h p.i., reaching 10^7,95^ PFU/mL and 10^7,82^ PFU/mL respectively.

Moreover, the same samples were used to measure the cells number and diameter, showing different trends for both cell parameters (Fig. 2b, c) and they were also assayed with Western blot on both supernatant and pellet of the Sf9 infected culture.

**Fig. 3:**
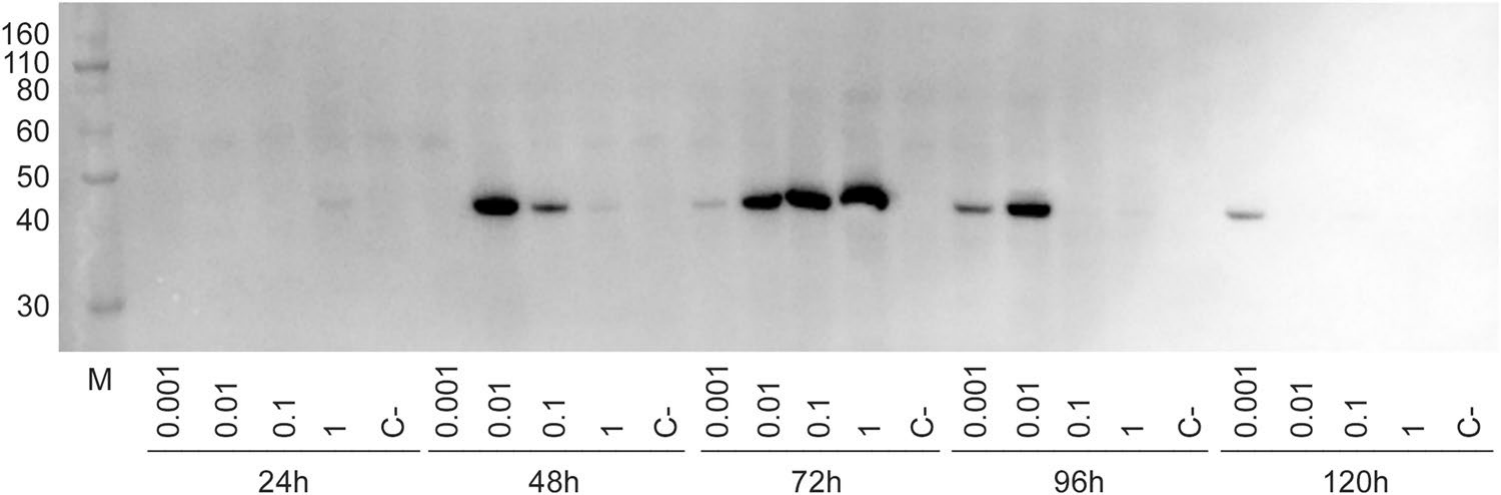
Western blot detection of recVP7 using anti-V5 antibody. Sf9 cells were infected with recombinant baculovirus at different MOI (0.001, 0.01, 0.1, 1) and after harvesting them by centrifugation at 24, 48, 72, 96 and 120 h, the samples of supernatant were checked in WB. Supernatant of uninfected cells was negative control and it is indicated as C−. The assay was performed denaturing the samples at 70 °C for 10′. *M* molecular weight marker, fragment sizes are measured in *kDa*

A strong reaction with anti-V5 antibody, consisting of a band at ~ 40 kDa, corresponding to the molecular weight of the monomeric form of BTV VP7, was revealed on supernatant samples at 48, 72, 96 h (Fig. 3). Results showed that the highest BTV-2 recVP7 expression was obtained at 72 h with MOI 1 and a similar result was obtained at 72 h with MOI 0.1 and at 48 h with MOI 0.01. Western blot on cell pellet was not evaluable (data not shown).

Combining cell viability and BTV-2 recVP7 expression results, for large scale production we chose to infect Sf9 cell culture with viral MOI 0.1, harvesting the infected culture at 72 h p.i. for subsequent protein purification. In particular, we infected 1.5 × 10^6^ Sf9 cells/mL in 1.5 L growth medium with MOI 0.1 of viral stock and then we harvested at 72 h p.i. the infected cell pellet, not discarding the supernatant, rather storing it at 4 °C for subsequent IMAC.

### Purification of BTV-2 recVP7 by Affinity Chromatography

BTV-2 recVP7 was produced at large scale and it was purified from both infected Sf9 cell pellet and supernatant.

**Fig.4:**
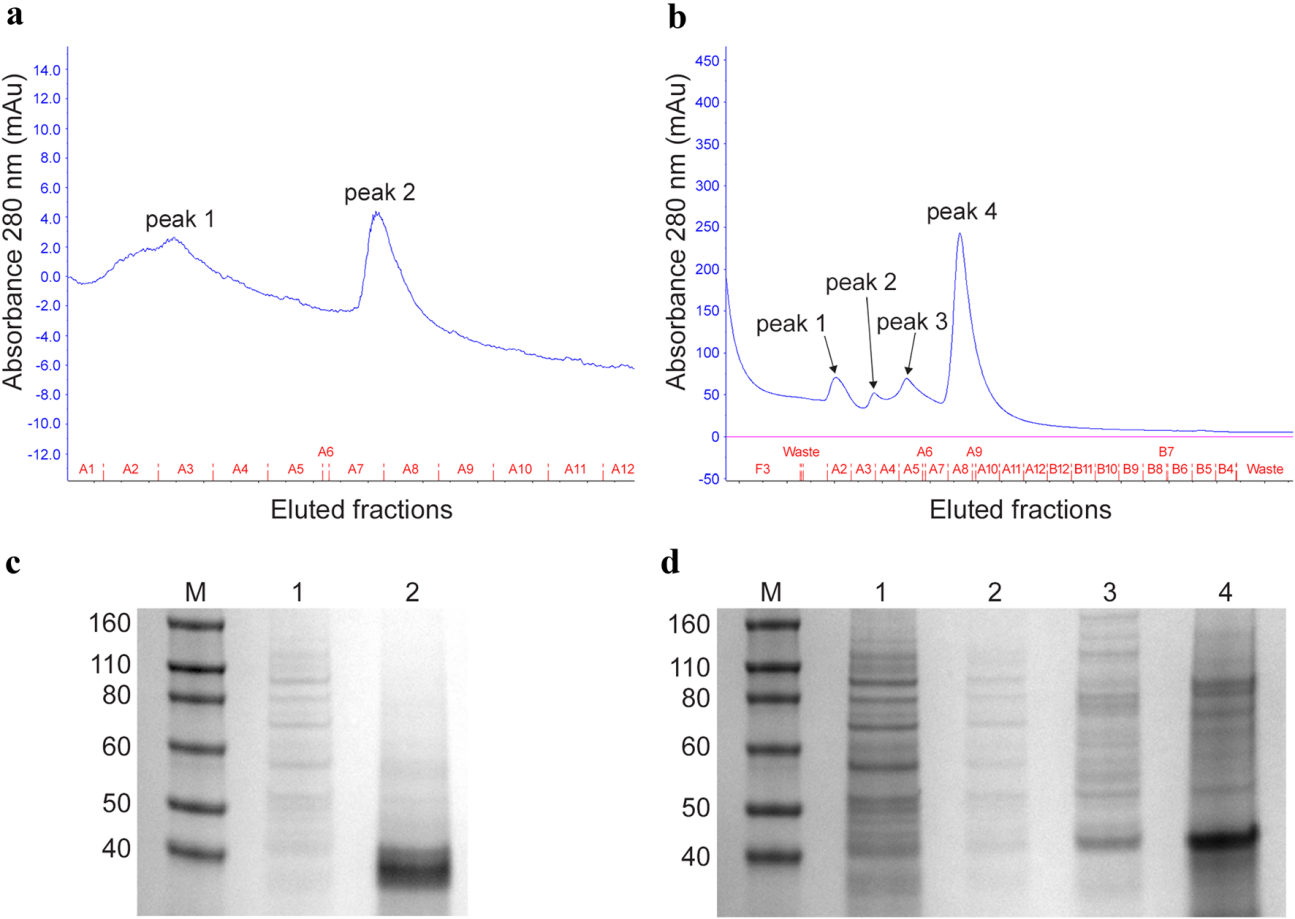
**a**–**d** Chromatograms of recVP7 purified by IMAC from infected Sf9 cell pellet (**a**) and infected Sf9 culture supernatant (**b**) and Coomassie staining of SDS-PAGE of all the eluted fractions from IMAC performed on the infected Sf9 cell pellet, after the ammonium sulphate precipitation (**c**) and on the infected Sf9 cell supernatant (**d**). **a**, **b** All the eluted fractions of the loaded samples are represented on *x* axis, and their respective absorbance values, measured at 280 nm (mAU), are reported on the *y* axis. **a** Peak 1 corresponds to the wash step to remove unknown contaminating proteins; peak 2 corresponds to elution step of the specific protein recVP7. **b** Peaks 1–3 correspond to the wash step of unknown contaminating proteins; peak 4 corresponds to elution step of the specific protein recVP7. **c** Lane 1 represents peak 1, fractions A2–A5 (**a**), that is the wash step with 20 mM imidazole, lane 2 represents peak 2, fractions A7-A9 (**a**), corresponding to the elution step with 250 mM imidazole, and containing the recVP7. **d** Lanes 1, 2 and 3 represent respectively peak 1, 2 and 3 of fractions A2–A6 (**b**), that is the wash step with 20 mM imidazole, lane 4 represents peak 4, fractions A7-A11 (**b**), corresponding to the elution step with 250 mM imidazole, and containing the recVP7. *M* molecular weight marker, fragment sizes are measured in kDa

As shown in chromatograms resulting from IMAC, the recVP7 that was first purified from the pellet by ammonium sulphate precipitation, had an extremely low absorbance at 280 nm, expressed in mAu value, almost near to 0, as shown in the peak 2, fractions A7-A9 (Fig. 4a). On the other hand, the recVP7 purified from the supernatant only by the one-step IMAC, was represented by peak 4, fractions A7–A11, and had a higher absorbance, reaching near to 250 mAu (Fig. 4b). The other peaks, mostly present in the supernatant sample (peaks 1–3, Fig. 4b), were considered weakly bound contaminants and were washed away with 20 mM imidazole.

Bradford assay confirmed that the purification of recVP7 from pellet and supernatant samples gave different yields: total protein concentration obtained from pellet was approximately 1 mg, whilst from supernatant reached 15 mg. The recVP7 derived from both pellet and supernatant, purified by IMAC, was then checked in SDS-PAGE and Western blot, and results are described in the next section.

### SDS, Semi-native PAGE and Western Blot

BTV-2 recVP7 recovered from infected Sf9 pellet immediately after the ammonium sulphate precipitation, was tested with SDS-PAGE and Western blot under denaturing conditions, using both anti-V5 HRP antibody and MAb anti-BTV. Results showed the presence in SDS-PAGE of a few bands of different non-specific molecular weight and the absence of specific reaction with MAb anti-BTV and anti-V5 antibody in Western blot (data not shown). As the level of BTV-2 recVP7 from pellet after the lonely ammonium sulphate precipitation were below visualization, maybe due to a dilution of the sample which was too high and to a poor grade of purification, we further purified the protein by IMAC and checked it again by SDS-PAGE. The recVP7 purified from infected Sf9 pellet, using firstly ammonium sulphate precipitation and then using IMAC, corresponds to elution peak 2 and fractions A7-A9 (Fig. 4a) as described in the previous results, and showed in its electrophoresis profile in Fig. 4c (lane 2). The peak 1, corresponding to the wash purification step, was also analized in SDS-PAGE (Fig. 4c, lane 1). The recVP7 purified from the pellet, peak 2 of the Fig. 4a had a high purity level with a major band of ~ 40 kDa, corresponding to the molecular weight of BTV-2 recVP7 monomeric form. On the same gel we loaded the BTV-2 recVP7 purified from the supernatant (Fig. 4b, d) and we identified (Fig. 4d, lane 4) a predominant band at ~ 40 kDa, corresponding to the molecular weight of the monomeric VP7, one minor band at ~ 80 kDa, corresponding to the molecular weight of the dimeric VP7. The sample of the lane 4 represents the elution peak 4 (Fig. 4b), fractions A7–A11.

The peaks 1, 2 and 3 are the eluted fractions of the wash step (Fig. 4b) and they were analyzed in SDS-PAGE, lanes 1, 2 and 3 (Fig. 4d) showing many no specific bands.

**Fig. 5:**
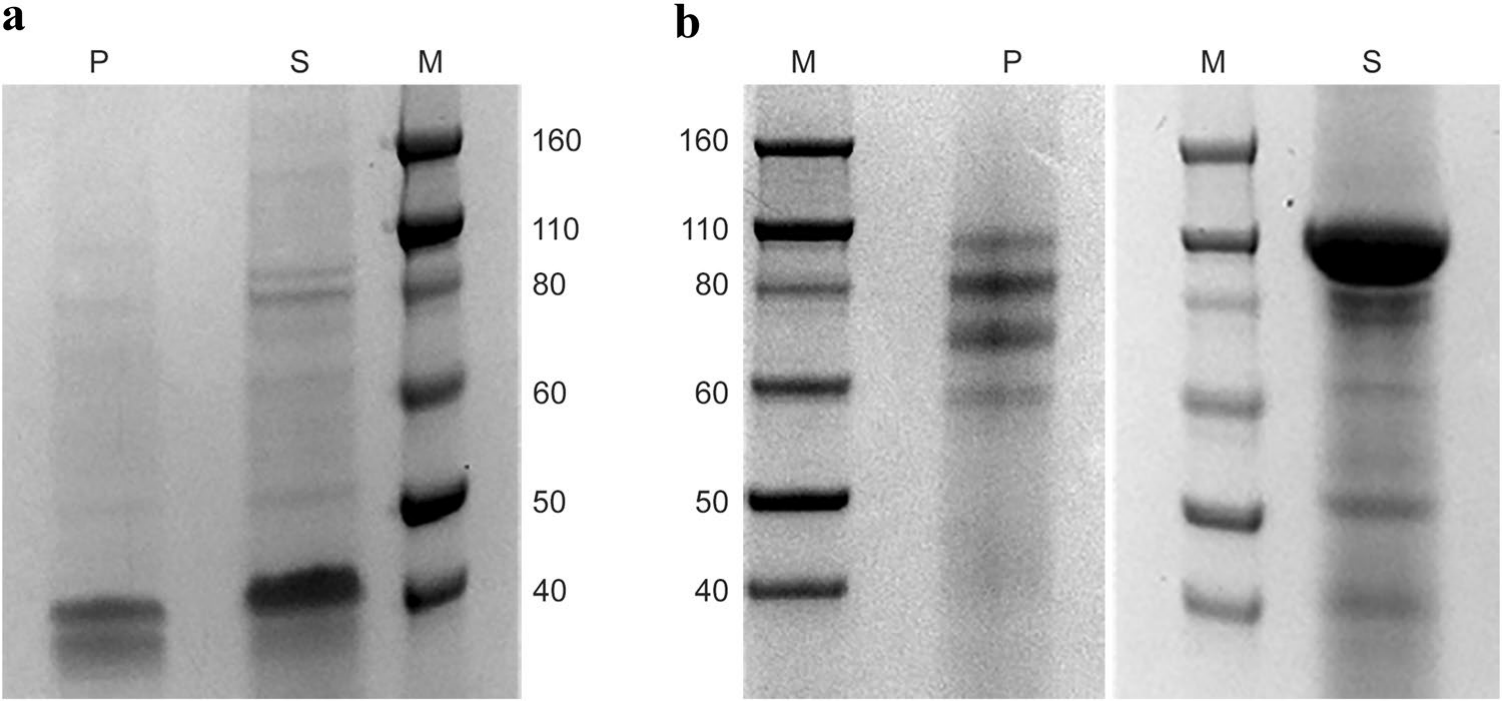
**a**, **b** Coomassie staining of SDS-PAGE (**a**) and semi-native PAGE (**b**) of recVP7 purified from the pellet (P) and from the supernatant (S) of infected Sf9 cells. **a** The recVP7 purified from P and S was denatured at 70 °C for 10′ before loading onto the gel. **b** The same samples of recVP7 purified by IMAC from P and S, previously checked by SDS-PAGE, as described in (**a**), were loaded onto the gel without heat treatment. *M* molecular weight marker, fragment sizes are measured in kDa

We compared the recVP7 purified from pellet and supernatant using both the SDS-PAGE and the semi-native PAGE (Fig. 5a, b) and the molecular weight observed in the sample of purified protein (Fig. 4c, d lanes 2 and 4 respectively) were confirmed (Fig. 5a).

The semi-native PAGE revealed the presence of bands ranging from ~ 110 to 60 kDa in the pellet sample, while the supernatant sample was represented by a major band at ~ 110 kDa, close to the molecular weight of the trimeric form of VP7, followed by one band at ~ 80 kDa, one band at ~ 40 kDa, corresponding to the molecular weight of dimeric and monomeric form of VP7 respectively, and some other bands of different molecular weight (Fig. 5b).

**Fig. 6:**
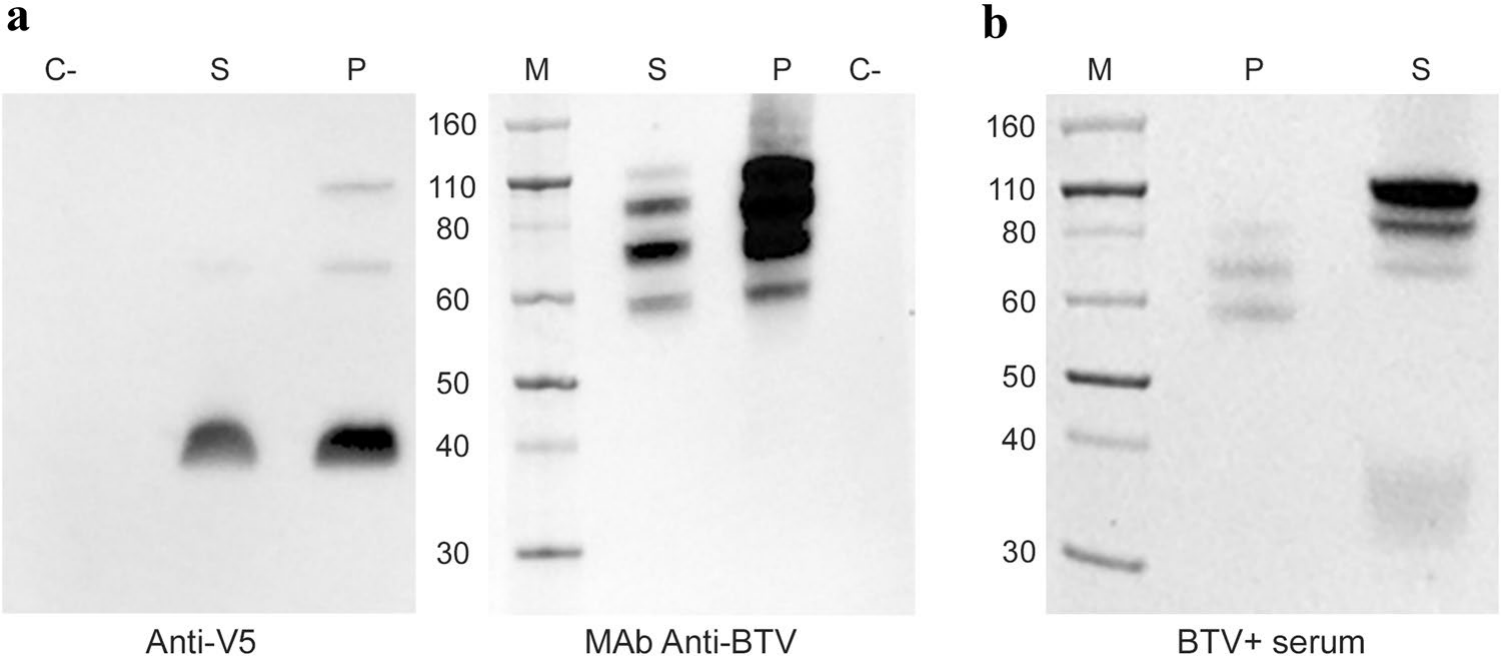
**a**,** b** Western blot detection of recVP7 purified from pellet (P) and from supernatant (S) of infected Sf9 cells. All the samples were analyzed under denaturing conditions, i.e. after performing a denaturation at 70 °C for 10′ before loading the samples onto the gel. **a** The bands show the reaction between the anti-BTV MAb, anti-V5 antibody, both conjugated with HRP, and the recVP7. **b** The recVP7 purified from both P and S was also checked for reaction with a positive bovine serum, detected using anti-bovine IgG secondary antibody. *M* molecular weight marker, fragment sizes are measured in kDa

To test the affinity of BTV-2 recVP7 produced at large scale with in house produced MAb anti-BTV [28] and to confirm the identity of our protein with the MAb anti-V5, we performed Western blot (Fig. 6a). The results show that BTV-2 recVP7 purified from the pellet binds to the MAb anti-BTV, generating 4 bands ranging from ~ 60 to 110 kDa. The BTV-2 recVP7 purified from the supernatant showed a band’s pattern similar to the pellet but with a different proportion amounts between the different oligomeric forms. In both samples, the MAb anti-BTV was unable to recognize the VP7 monomeric form predicted at ~ 40 kDa, in contrast to the anti-V5 antibody that recognized not only a predominant band at ~ 40 kDa, but also two bands of minor intensity at 70 and 110 kDa, in the pellet sample. Results regarding affinity of BTV-2 recVP7 with anti-V5 antibody confirm the previous Western blot results of unpurified BTV-2 recVP7, taken from pellet/supernatant of P1/P2 infected cells (Fig. 1).

We tested an experimentally infected BTV positive bovine serum in Western blot (Fig. 6b), under denaturing conditions, and we identified weak bands at ~ 60–80 kDa in recVP7 purified from the pellet, in contrast to the strong reaction with recVP7 purified from the supernatant, whose bands were visible at ~ 80–110 kDa. Moreover, the bovine positive serum was also able to detect a smear between 30 and 40 kDa, that could be a degraded form of recVP7 or a faint aspecific reaction.

**Fig. 7:**
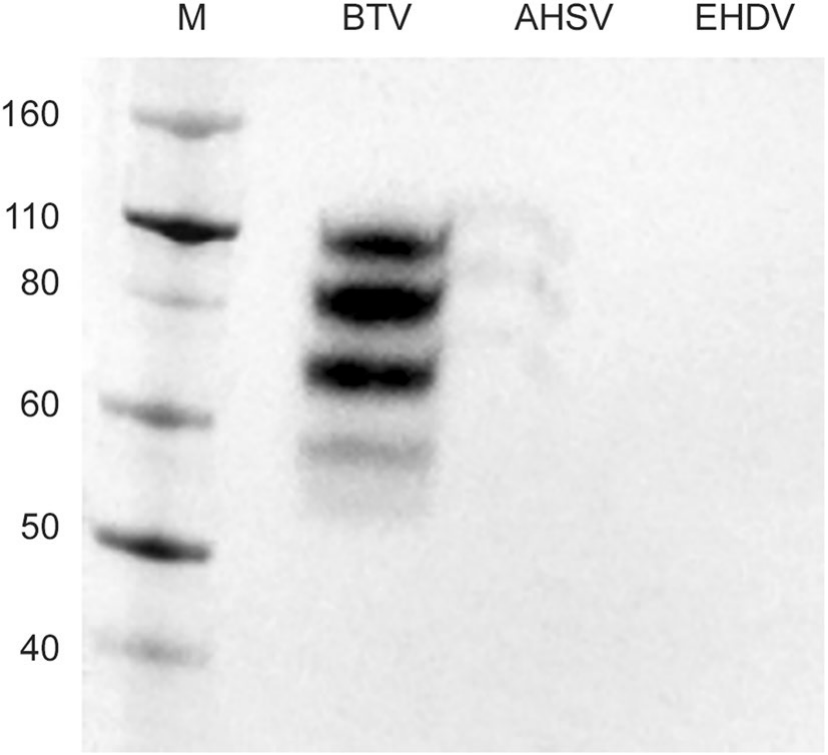
Analysis of the anti-BTV MAb cross-reactivity using Western blot. Samples of recombinant VP7 from BTV, AHSV and EHDV were tested for reaction with HRP-conjugated anti-BTV MAb. *M* molecular weight marker, fragment sizes are measured in kDa

The MAb anti-BTV showed elevate specificity, recognizing only BTV-2 recVP7 and showing no cross-reactivity with the recVP7 of AHSV and EHDV (Fig. 7).

### Competitive-ELISA

**Fig. 8:**
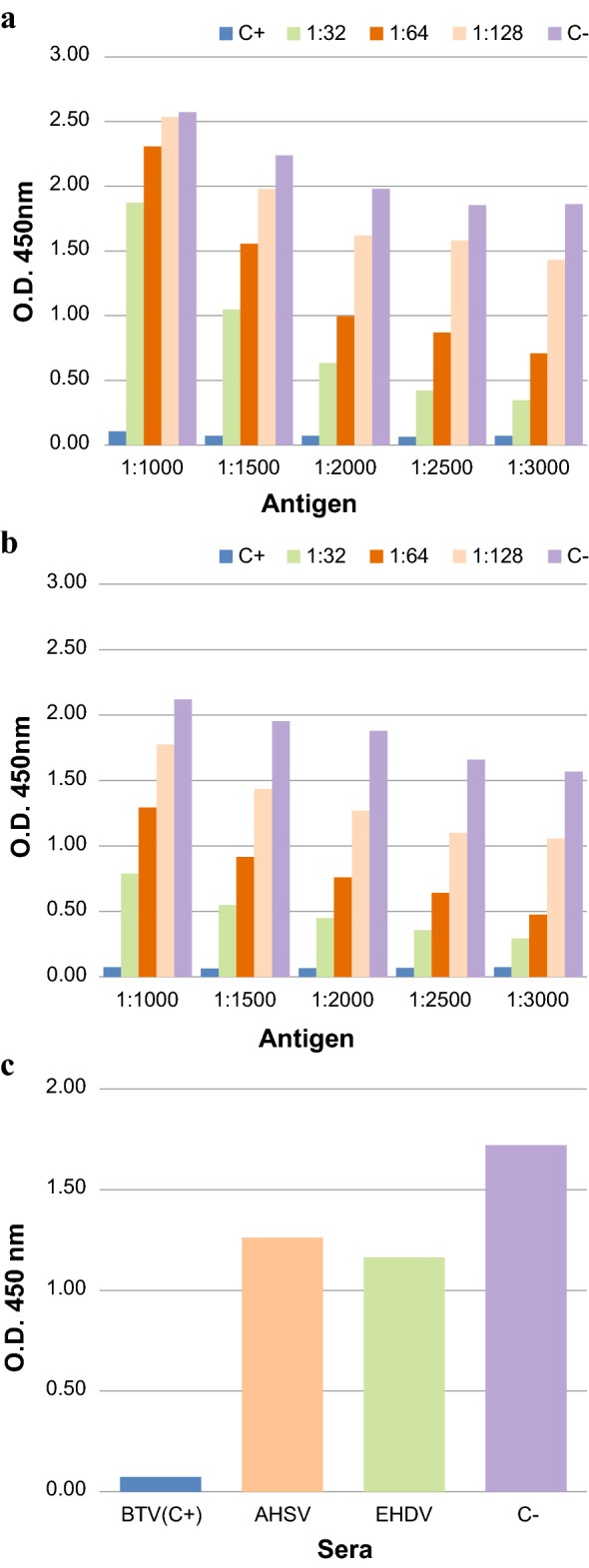
**a**–**c** Graphical representation of c-ELISA results for recVP7 purified from the supernatant (sVP7, **a**) and from the pellet (pVP7, **b**). Different pVP7 and sVP7 dilutions were used. The ability of the MAb to interact with recombinant proteins was measured at O.D. 450 nm. Undiluted BTV positive serum was used as positive control ( C +); BTV negative serum was used as negative control ( C−); BTV positive serum was diluted  1:32,  1:64 and  1:128) **c** The recVP7 purified from supernatant (sVP7) was used to test, by c-ELISA, the cross-reactivity with BTV (C+), AHSV and EHVD positive sera. C− was negative serum for BTV, AHSV and EHDV

For the analysis of the results, we considered the O.D. obtained using MAb at 1:300,000 dilution. In all c-ELISA performed, the positive control showed the O.D. values below 0.1. The O.D. values of negative control ranged from 2.12 to 1.57 and from 2.57 to 1.86 at the different dilutions of pVP7 and sVP7 respectively (Fig. 8a, b). Moreover, testing different dilutions of BTV positive serum, at all dilutions of pVP7 and sVP7 antigens the O.D. values showed the same trend for pellet and supernatant with lower values in the pellet sample.

When using pVP7 and sVP7 diluted 1:2000, the lowest variability of the O.D. values was observed between the two antigens, (Fig. 8a, b), independently of the BTV positive serum dilutions.

Immunoenzymatic assay was performed in order to verify the specificity of interaction of BTV-2 recVP7 with BTV positive sera, testing undiluted positive sera of AHSV and EHDV on a plate activated with sVP7 diluted 1:2000. The results showed that the O.D. values were higher than 1.00 for both AHSV and EHDV sera, in contrast to values obtained with BTV positive control, that was below 0.1 (Fig. 8c).

**Fig. 9:**
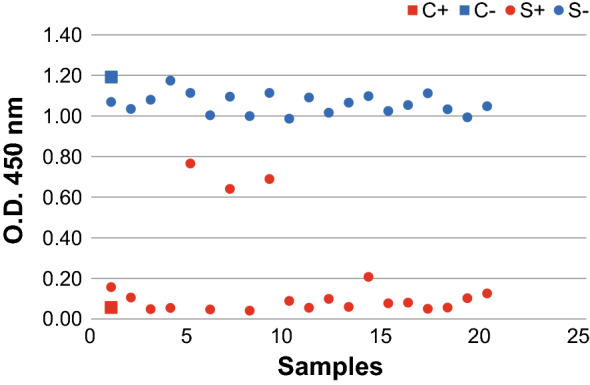
c-ELISA of BTV sera. 20 BTV positive and 20 BTV negative sera ( S+ and  S− respectively) were tested. Positive BTV serum ( C+) and negative BTV serum ( C−) were used as control

C-ELISA performed on 20 positive and 20 negative sera, showed a difference between the BTV positive and negative samples (Fig. 9). All the negative sera had O.D. values ranging from 1.00 to 1.2. The BTV positive sera had O.D. values below 0.2, except that 3 out of 20 that had values corresponding to 0.77, 0.64 and 0.69.

## Discussion

VP7 protein is the preferred choice for developing group-specific serological assays for BTV diagnosis, due to its highly conserved sequence and antigenicity between different strains [19]. In this study, we used a baculovirus system for the expression of recombinant VP7 of BTV-2, to be further evaluated as diagnostic reagent in c-ELISA.

Recombinant VP7 was successfully obtained and it was first produced at small-scale in order to assess the best conditions for its expression. BTV-2 recVP7 expression level was checked at different TOH of Sf9 cells infected with recombinant baculovirus at different MOI. The titration of the recombinant baculovirus was performed using the Sf9 Easy Titer cells [29], that allowed us to do a rapid and accurate titration, unlike the plaque assay [34, 35] that is well known to be a time consuming and labor intensive technique [36].

The highest viral titer was present at 48 h with MOI 1 (Fig. 2a), but cell viability was clearly lower than cell viability present at 72 h and MOI 0.1 (Fig. 2b).

The use of MOI 0.1 and 72 h as TOH in BTV-2 recVP7 production protocol, helped us to minimize the risk of protease action on BTV-2 recVP7. The influence that cellular proteases can have on recombinant protein expression as already been object of investigation [37, 38]. In particular, it was observed that host cell proteases may influence the final yield of the recombinant protein, especially after 72 h p.i., the moment when a substantial decline in product yield has been observed in many reports [39, 40]. It was also reported a correlation between the used MOI and the proteases, suggesting that a high MOI is related to strong protease activity. It was not excluded a role of cell lysis, then cell viability, in the process of degradation of the recombinant protein [37].

In our experiments, we used protease inhibitors only when manipulating the infected Sf9 cell lysate, immediately before the ammonium sulphate precipitation. We did use protease inhibitors neither in the cell culture nor in the supernatant, after harvesting the cells, because of the quantity of protease inhibitors to be used in 1.5 L of cell culture, according the manufacturer instructions (Roche), it would be too expensive, since the choice of the correct MOI and TOH lead us to have no product loss and/or degradation. The expression of recVP7 at the different MOI and TOH, as shown in Fig. 3, is represented by a weak band at 48 h and MOI 1, despite the high titer, thus confirming that low cell viability (Fig. 2b) affects the expression of the recVP7. We can also observe a progressive decrease in recVP7 expression with the increasing time post infection, so TOH.

Furthermore, we can observe that the recVP7 expression at 48 h and 0.01 MOI was quite similar to the expression at 72 h, 0.1 and 1 MOI (Fig. 3), even if the viral titer and cell diameter were lower at 48 h and 0.01 MOI (Fig. 2a–c).

Finally, MOI 0.1 and 72 h as TOH were chosen for the large scale production of BTV-2 recVP7, because of these values correspond to the best viral and cellular parameters, i.e. viral titer, good cell viability and cell diameter used as infection indicator (Fig. 2a–c).

Once defined optimal conditions for the expression of recVP7, its production was scaled-up, to obtain high yield of recombinant protein purified by IMAC. The IMAC used to purify the recombinant protein from the supernatant, offered several advantages as the automatization of the process which allows to speed the process and improve its standardization [23, 41–43]. While high yield of recVP7 was obtained from a single-step purification of the supernatant by IMAC, the purification of recVP7 from the pellet, by ammonium sulphate precipitation and subsequently by IMAC, did not provide similar results, confirming that the BTV-2 recVP7 was mostly released from the transfected cells as previously described [26, 44]. In addition to these last scientific reports that describe the opportunity of VP7 isolation from cell culture supernatant, the most part of reported experiments are based on the isolation of recombinant VP7 from infected Sf9 cell lysate [9, 18, 25]. In some cases, the recombinant protein was not even purified [26, 27, 40] and the yield of recombinant protein obtained at the end of the production process was not always mentioned, as well as the method of protein quantification [23–25]. Some authors reported the yield obtained, that was characterized, from one author to the other, by an extreme variability [9, 20, 40, 44, 45]. Since we preferred to optimize the recVP7 production from supernatant of infected Sf9, because of its easy, rapid and costless production, we can not compare the yield of our recVP7 with the other ones previously published, these last being mostly related to recombinant proteins isolated from cell lysates.

In our study, when analyzing the recVP7 isolated from infected Sf9 pellet and from supernatant, we highlighted the difference in terms of yield: the recVP7 purified from the pellet of 1.5 L of infected Sf9 culture gave us only about 1 mg, while we were able to obtain almost 15 mg of recombinant recVP7 from the supernatant.

The purity, the positive reaction with the MAb anti-BTV and the performance in c-ELISA, were other factors that we had to consider, in addition to the yield, in order to produce the recVP7. After performing IMAC, we obtained a highly purified recVP7, from both pellet and supernatant, with the difference that the supernatant was subjected to only one-step purification procedure, when compared to the pellet one. Moreover, the 6xHis-tag in N-terminal, while necessary for the purification process, did not interfere in the reaction between the recVP7 and the MAb anti-BTV, as showed by the immunoassays that have been performed to characterize the protein [23]. The recVP7 purified from both pellet and supernatant was tested with the MAb anti-BTV produced in house [28] in Western blot and in c-ELISA. When analyzing the Western blot results, we can observe that the binding between the MAb anti-BTV and the recVP7 was not represented by a single band, such as the expected single band at 40 kDa of the monomeric VP7, but it was observed the presence of bands of different molecular weight, mainly ranging from 60 to 110 kDa.

This finding can be explained by the temperature that we used for the denaturation step before loading the samples onto the NuPAGE gels. In particular, we denatured the samples at 70 °C, as suggested by the manufacturer instructions (Invitrogen), and we also tried to denature samples at 95 °C (data not shown). The recVP7 was visible in SDS-PAGE when denatured at both temperatures as a 40 kDa band and, specifically when denatured at 70 °C some other bands at higher molecular weight, thus suggesting that the protein can be present at this temperature in different conformational states due to an incomplete denaturation, as already reported in literature [25, 46–48].

While in SDS-PAGE we were able to visualize the recVP7 using both denaturation temperature (70 °C and 95 °C), the reaction between the recVP7 and the MAb was visible using only 70 °C as denaturation temperature, confirming that denaturation of recVP7 at this temperature is incomplete, the our MAb anti-BTV recognize only oligomeric forms of the recVP7 and that the MAb anti-BTV binds to a conformational epitope of recVP7. On the same samples, we performed semi-native PAGE (Fig. 5b), and we observed that, even without any heat denaturation, there was no band present at exactly 120 kDa, but rather there is a major band at 110 kDa and other bands of different lower molecular weight. Similar results were already reported in literature [25, 46], where recombinant VP7 was analyzed by trimerization assays, using 37–100 °C and R.T.–100 °C as denaturation temperatures [25, 46].

The presence of bands at different molecular weight in semi-native PAGE in our study (Fig. 5b) and also in Monastyrskaya et al. and Limn et al. [25, 46], suggests that a minimal denaturation of the samples occurs, probably due to the action of the reducing agent of the sample loading buffer and of the SDS contained inside the gel. We demonstrated the specificity of reaction between the recVP7 and MAb anti-BTV checking the cross-reactivity with other Orbiviruses recombinant VP7, using Western blot and the results (Fig. 7) showed the bands only in the BTV2 recVP7 sample.

We analyzed the recVP7 purified from both pellet and supernatant in Western blot using also a BTV positive serum (Fig. 6b), and the results showed a bands pattern similar to the MAb one, even if, using the positive serum, the band intensity of the recVP7 purified from the supernatant was much higher than the intensity related to the recVP7 from the pellet. This result was of critical importance, because, in c-ELISA, antibodies of BTV positive sera go in competition with the MAb anti-BTV for the binding to the recVP7.

After performing Western blot, we tested the recVP7 in c-ELISA, in order to evaluate the recombinant protein object of this study as a useful reagent for diagnostic purpose. The results of c-ELISA, showed just a little difference in O.D. values between the recVP7 purified from the pellet (pVP7) and from the supernatant (sVP7), probably due to the slightly higher purity grade of pVP7.

Highly purified protein has a key role for production and validation of diagnostic assays [49, 50], but, on the other hand, the recVP7 isolated from the infected Sf9 pellet had to be subjected to the several manipulations during purification, thus going consequently to the detriment of recombinant protein yield. Even if the pVP7 had a higher purity, the sVP7 reached the same performance of pVP7, in particular at 1:2000 antigen dilution (Fig. 8a, b).

BTV-2 recVP7 purified from supernatant was then used to check any cross-reactivity with antibodies of related *Orbiviruses*, such as EHDV and AHSV, and a high degree of specificity was demonstrated (Fig. 8c). Finally, the recVP7 purified from supernatant was successfully used to discriminate BTV positive bovine sera from negative ones (Fig. 9).

In order to develop an immunoenzymatic assay for diagnostic purpose, we considered different critical elements, i.e. the purity and yield of the recombinant protein, performance in c-ELISA and, not less important, the time spent and the costs for purification method.

The choice of producing a purified recombinant protein allowed us to overcome problems derived from the use of whole virions as antigen [28, 49–51], used in previous ELISA assays.

The use of unpurified viral antigen is usually related to less stable protein preparation, due to the presence of extraneous proteins with enzymatic activity or to the characteristic of viral proteins to assemble into virus-like structures, leading to uncontrolled aggregation with extraneous proteins or to self-aggregation [18, 23].

The use of the infected Sf9 supernatant allowed us to produce a high quantity of recombinant protein with high purity level, obtained by an easy one-step procedure, i.e. the lonely IMAC purification, rather than the multistep purification from the pellet.

Looking at the c-ELISA experiments, these last showed similar results using the same concentration of recVP7 purified from both pellet and supernatant, therefore we decided to use the recVP7 protein purified from supernatant, not only in the single last experiment for discriminating the BTV positive from negative sera, but also in a final view of large-scale production, where the recVP7 purified from supernatant fits better in terms of yield, costs and ease of production. In fact, the high quantity of recVP7 that we obtained, using the supernatant as source of recombinant protein, would allow to produce a high number of diagnostic kits that could be marketed and used to detect the presence of anti-BTV antibodies in animal sera.

In order to obtain the results showed in this study, we produced in our laboratories different lots of recVP7 purified from supernatant and the yield and the degree of purity were always confirmed. These preliminary data lay the foundations for the conduction of further extensive studies, aimed to test the reproducibility and reliability of the methods here described for production and purification of BTV-2 recVP7 from cell culture supernatant and to assess the recVP7 stability over time. The development and validation of c-ELISA for BTV diagnosis, using recVP7 purified from supernatant represents the final goal of this study.
